# Evaluation of Telephone Visits in Primary Care: Satisfaction of Pediatricians and Family Physicians and Their Perceptions of Quality of Care and Safety

**DOI:** 10.3390/healthcare12020212

**Published:** 2024-01-15

**Authors:** Tamar Shalom, Osnat Bashkin, Alexander Gamus, Yoram Blachar, Shlomit Yaron, Doron Netzer, Ayelet Nevet, Gil Lavie

**Affiliations:** 1 Department of Health Systems Management, The College of Law and Business, Ramat Gan 52520, Israel; alexgamus@gmail.com (A.G.); yoramblachar@gmail.com (Y.B.); 2 Department of Public Health, Ashkelon Academic College, Ashkelon 78211, Israel; 3 Division of Community Medical Services, Clalit Health Services, Tel Aviv 62098, Israel; yshlomit@clalit.org.il (S.Y.); doronne@clalit.org.il (D.N.); ayeletn@clalit.org.il (A.N.); 4 Adelson School of Medicine, Ariel University, Ariel 40700, Israel; 5 Branch of Planning and Strategy, Clalit Health Services, Tel Aviv 62098, Israel; gillav@clalit.org.il; 6 Ruth and Bruce Rappaport Faculty of Medicine, Technion, Israel Institute of Technology, Haifa 31096, Israel

**Keywords:** telemedicine, telehealth, telephone visit, safety, quality, satisfaction, service assessment

## Abstract

Telehealth has accelerated since the outbreak of the COVID-19 virus. As telephone visits become more common, it is important to examine the challenges involved in using this modality of care. In this study, we examined family physicians’ and pediatricians’ perceptions regarding three aspects of the use of telephone visits: quality of care, safety of care, and physicians’ satisfaction. A total of 342 family physicians and pediatricians responded to an online survey. Respondents were asked to rate their degree of agreement with 17 statements inquiring about quality, safety, and satisfaction with telephone visits on a Likert scale ranging from 1 (strongly disagree) to 5 (strongly agree). This was followed by in-depth interviews between January and April 2023 with 26 physicians. Participants expressed satisfaction (3.66 ± 0.80) with the use of telephone visits and lower assessments of safety (3.03 ± 0.76) and quality (2.27 ± 0.76) of care using the telephone modality. Eighty percent of the respondents think combining a face-to-face visit with a telephone visit is recommended, and 51% noted that the inability to examine patients closely affects and impedes a physician’s decision making. Most interviewees indicated that telephone visits are safe only with former patients they had already seen in the clinic. The findings shed light on the perceptions of family physicians and pediatricians regarding telephone visits. The lower assessments of quality and safety compared to the assessment of satisfaction underscore the need for careful use of telephone visits in healthcare. A proper and balanced selection of patients, implementing technological upgrades to the modality, and performing patient education practices are recommended.

## 1. Introduction

Telemedicine delivers healthcare services using information and communication technology to remotely diagnose, treat, and prevent disease or injuries, conduct research, and provide evaluations and education for healthcare providers and their communities [1]. The COVID-19 pandemic changed patterns of operation in the healthcare system, including a transition to providing medical care through remote encounters. This proved central in preventing the transmission of the coronavirus and protecting both patients and service providers [2].

Telemedicine presents an opportunity to overcome many obstacles, including those of geography and logistics. According to a recent review, family physicians were the healthcare providers who made the most use of telemedicine services such as telephone visits. Telephone encounters can provide a means of following up with patients and monitoring disease, empowering patients, guiding patients, and providing quick and easy access to medical resources [3]. Remote care modalities in pediatric medicine, such as telephony, videoconferencing, and remote monitoring, have increased recently. Remote consultation over the telephone is a particularly popular modality for patient care among patients [4,5]. In a systematic review of telemedicine in pediatrics, Shah and Badawy (2021) demonstrate the distribution of various telemedicine approaches, such as videoconferencing consultations (45%), smartphone-based interventions (27%), telephone counseling (18%), and telemedicine-based screening visits (9%). The telephone modality is considered a common alternative form of care to face-to-face consultation in clinical settings [6].

Previous evidence showed outcomes involving symptom management, quality of life, satisfaction, medication adherence, visit completion rates, and disease progression among patients using telehealth technologies, were similar to, or better than, the outcomes of the control groups [5]. Many acute pediatric conditions can be managed safely and effectively through telemedicine, mainly when remote physical exam equipment is used [7].

While telephone consultations have many benefits, some concerns need to be addressed. A study conducted in Israel before the COVID-19 pandemic demonstrated several challenges for physicians, including diagnosing from a distance, treating unfamiliar patients, call urgency and loads, technical obstacles, and a “moral conflict” between the desire to meet parents’ expectations and maintaining standards of care [8]. Nonetheless, the use of telemedicine during the pandemic and beyond has become prevalent, and additional challenges must be met to successfully expand telephone consultation as a modality of remote care [9].

According to a survey carried out in Israel that included 2536 respondents between October 2021 and March 2022, it was found that 19% reported that they contacted their family doctor during their last visit by telephone or video visit. It total, 44% of the respondents reported using telemedicine services (telephone visit, video visit, or chat) in the last year [10]. Data based on all visits to primary care in Clalit Health Services, the biggest HMO in Israel with more than 4 million patients, show that the share of visits provided remotely started at 5% before COVID-19, peaked at 40% during the first COVID-19 lockdown in April 2020, and stabilized to 20% post lockdown around June 2021 when the COVID-19 rate in Israel was very low [11]. Based on data that include all visits to pediatricians at Maccabi Health Services, the second largest HMO in Israel with approximately 2 million patients, telephone visits accounted for 0% of pediatrician visits before the pandemic, 17% of pediatrician visits during the first lockdown period, and 19% of pediatrician visits one month after the lockdown in May 2020 [5]. According to OECD data, the average number of annual doctor consultations among 12 European countries was 5.1 in-person consultations and 1.4 teleconsultations, meaning that about 22% of all consultations were teleconsultations [12]. In the US in 2021, according to the National Center for Health Statistics (NCHS), 37% of the adult population received remote health care in the last 12 months (with higher use by women and higher use for older ages) [13]. According to a national survey in the US conducted in 2021 and 2022, 22.5% reported the use of telehealth services (audio only or video) within the last 4 weeks [14]. Although the data presented reflect the use of telephone visits as part of the use of telemedicine services, it is possible to see a similar or higher use of telephone visits in Israel compared to other Western countries. It appears that telemedicine tools such as telephone visits have become an integral part of our lives and will continue to be used in the healthcare system. Previous studies investigated the use of telemedicine and telephone visits following COVID-19, in Israel and Western countries. Most studies focus on the frequency of use and patients’ satisfaction with telemedicine but do not deal with doctor’s satisfaction with the use of telephone visits and their perceptions of the quality and safety of its use. Consequently, it is essential to examine physicians’ perceptions and concerns regarding telephone visits and engage physicians in development processes to improve and enhance the acceptance of telemedicine. The current multi-methods study aimed to assess physicians’ satisfaction with telephone visits, evaluate the quality and safety of the telephone modality as perceived by pediatricians and family physicians, and generate recommendations to increase the efficiency and acceptability of this service. In mixed-method research, quantitative and qualitative methods are employed together to create a research outcome stronger than either method alone [15]. This method enables exploring more complex aspects of human and social interactions, therefore gaining a deeper understanding of the perceptions of physicians toward the use of telephone visits.

## 2. Materials and Methods

This study utilized qualitative and quantitative assessment methods, including a survey and in-depth interviews among pediatricians and family physicians working in Clalit Health Services (Clalit), Israel’s largest health maintenance organization (HMO). The study was approved by the Ashkelon Academic College Ethics Committee (Approval #43-2022). Multi-methods research is a systemic approach to understanding the interaction of variables in a complex environment [16]. While quantitative assessment allows for exploring causal linkages among sets of data, qualitative assessment persuades through the rich description of perceptions and adds an understanding of the phenomenon that numbers alone cannot [15].

### 2.1. Population Sample

In total, 1000 pediatricians and 2500 family physicians working in Clalit were contacted by researchers, of which 342 responded to an online survey distributed via electronic mail (e-mail) (9.8% response rate) between January and April 2023. Two electronic e-mail reminders were sent one month and two months after the initial contact. The survey was closed in April 2023.

**Figure 1:**
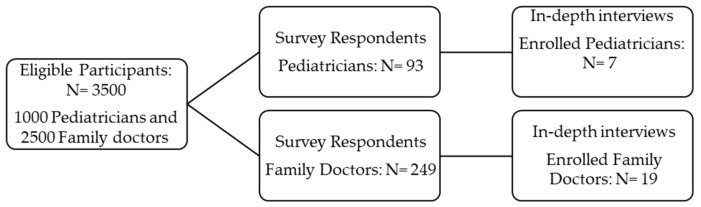
Flow diagram for study participants.

Interviewees were recruited using purposeful sampling. Purposeful sampling is a non-random sampling technique that uses specific criteria or purposes to select a sample [17]. The aim is to collect in-depth information from the right respondents. The inclusion criteria: pediatrician or family physician working in Clalit. We continued the interviews until theoretical saturation was reached. Signed informed consent was obtained for additional interviews, which were administered to 26 participants of the sample (7 pediatricians and 19 family physicians. See Appendix A) between March and April 2023. The interviews, lasting between forty minutes and an hour, were conducted by one of the authors (TS), who specializes in qualitative research methods in healthcare settings over the phone. Figure 1 describes the study participants in both quantitative and qualitative phases of data collection.

### 2.2. Research Tools

The survey developed by the researchers (see Supplementary File S1) was validated by two family physicians and one pediatrician using the content validation method. The content validation process included a readability test to determine whether the items or questions effectively represent the variables or constructs measured. Following their comments, ambiguous questions were clarified, and the questionnaire was evaluated in a piloted study. In the pilot, 12 participants including the study team and physicians responded to the survey. Respondents were asked for feedback related to the accuracy of the survey instructions and questions. The final questionnaire included 17 statements in three subsets: quality, safety, and satisfaction of telephone visits with patients. Four questions (3, 4, 5, and 6) were excluded from the classification as they represented perceptions of potential improvements and the usefulness of the telephone modality of telemedicine. Respondents were asked to rate their degree of agreement on a Likert scale ranging from 1 (strongly disagree) to 5 (strongly agree). Questions 8 and 10–12 have a reverse evaluation scale; therefore, the responses were transposed for uniformity with further assessment. The questionnaire also included sociodemographic factors such as gender, age, number of years of experience in medicine, number of years of experience in telephone visits with patients, the geographic area of work in Israel, and the physicians’ sector of the population (General, Arab, Ultra-Orthodox, which is a minority religious group characterized by strict adherence to the Jewish law of Halakha). In addition, participants were asked for their opinions regarding the situations in which telephone visits can be used safely. Table 1 presents the survey instrument validity assessment (Cronbach’s alpha of the complete survey was α = 0.87).

The interview guide was developed as a complementary tool to the survey (see Supplementary File S1). The topics that guided the question development were in line with the survey protocol as follows: (1) assessment of the quality of the telephone visit service, (2) challenges associated with the safety of the telephone visit service, and (3) physicians’ satisfaction and concerns related to the telephone visit service. The interview guide was validated using the content validation method with one pediatrician and one family doctor to ensure a smooth interview flow and verify comprehension of the questions. The in-depth interviews were semi-structured. The wording and order of the questions changed according to the interview dynamics to maintain continuity and encourage openness of the interviewees. No prior relationship existed between the interviewer and the participants.

### 2.3. Statistical Analyses

Internal consistency was assessed by applying Cronbach’s α coefficient to each subset and the overall instrument. In order to describe the distribution of the responses to the statements, Pearson’s product–moment correlations of corresponding subsets and intra-class correlation coefficients (ICC) were calculated. Student *t*-tests were employed to establish the differences between subsets. A one-way ANOVA test included post-hook analysis using the Tukey method. Finally, multiple linear regression models with perceptions toward quality, safety, and satisfaction as outcomes were tested. A statistical significance level of α = 0.05 was assumed throughout the study. All testing was performed using IBM SPSS Statistics for Windows, Version 25.0. Armonk, NY, USA: IBM.

The interviews were analyzed using a thematic analysis method based on grounded theory [18]. The analysis included incorporating deductive themes from the research topic and based on an exhaustive literature review of telemedicine, together with inductive themes that emerged from the data [19]. As part of the analysis of the transcripts, all interviews were read several times to obtain a thorough understanding of the data. Following the study’s objectives, researchers identified concepts, categories, and themes. Re-reading the transcripts prompted a reevaluation of the central themes by adding encoded quotes and examples. During the analysis, we conducted an ongoing internal quality audit, adapted from Mays and Pope [19]. The themes and quotes were translated and documented in English at the final stage. We used a standardized codebook to ensure the validity of the translations from Hebrew to English.

## 3. Results

### 3.1. Sample Characteristics and Perceptions towards Telephone Visits

Table 2 presents the survey participants’ demographic data. The mean experience for family physicians and pediatricians was 23 years (SD ± 11.4) and 25 years (SD ± 10.5), respectively. The experience with the telephone modality was 5 years (SD ± 6.5) for family physicians and 5 years (SD ± 6.2) for pediatricians.

Table 3 presents the means and standard deviations of the three research variables. No significant differences were observed between pediatricians and family physicians in perceptions of the quality, safety, and satisfaction of physicians regarding the use of telephone visits.

Table 4 presents the distribution of the responses. Anchor points 1 (strongly disagree) and 2 (somewhat disagree), and anchor points 4 (somewhat agree) and 5 (strongly agree) were combined to present the distribution. The survey population’s responses showed that 48.4% of the respondents think that telephone visits cannot replace a face-to-face meeting, and 43% think that the quality of care in telephone visits is not the same as the quality of care provided in clinic visits. Similarly, about 80% think combining a face-to-face visit to the clinic with a telephone visit is recommended. Half of the respondents believe telephone visits are unsafe for patients and may involve risks. Fifty-four percent mentioned that telephone visits shorten the duration of a visit compared to a face-to-face visit in the clinic. Half of the respondents noted that the inability to examine a patient closely affects the doctor’s decision making, making it difficult. Nevertheless, half of the respondents expressed satisfaction with telephone consultations. Fifty-two percent of the respondents agreed with the statement regarding whether transferring photos and documents would improve the quality of care, and 40.8% agreed with the statement regarding whether or not incorporating video recording during a telephone visit would improve the quality of service.

Table 5 presents the distribution of the responses to the question regarding the situations in which telephone visits can be used safely. In total, 222 of the participants responded to this question (64.9%). Most family physicians mentioned administrative care, routine actions, follow-up, and providing prescriptions as the situations in which telephone visits can be used safely. Pediatricians had similar opinions about the addition of not complicated cases.

### 3.2. Differences in Perceptions towards Telephone Visits

A one-way ANOVA test and post hoc test using the Tukey method were performed to examine differences in perceptions towards telephone visits among sociodemographic groups of participants. Analysis showed significant differences by population sector regarding the perceptions of safety and satisfaction in the use of telephone visits (F = 3.16, *p* < 0.05 and F = 4.11, *p* < 0.05, respectively). Ultra-Orthodox physicians expressed lower perceptions of safety compared to Arabs and the general population (mean 2.78 (SD ± 0.7), 3.22 (SD ± 0.6), and 3.02 (SD ± 0.8), respectively). In addition, ultra-Orthodox physicians expressed lower satisfaction compared to Arabs and the general population (mean 3.31 (SD ± 0.8, 3.86 (SD ± 0.8), and 3.66 (SD ± 0.8, respectively). Furthermore, significant differences were found in the perceptions of safety according to geographical area of work (F = 3.09, *p* < 0.05). Physicians who work in the central part of the country expressed lower perceptions of safety compared to those who work in the north and the south of Israel (mean 2.96 (SD ± 0.8) vs. 3.20 (SD ± 0.6) and 3.00 (SD ± 0.8), respectively). No differences were found among age groups and years of experience in perceptions toward the quality of care, safety, and satisfaction with telephone consultations. To examine predictors of perceptions toward quality of care, safety, and satisfaction with telephone consultations, multiple linear regression models were tested. No variable showed a significant contribution to the prediction. (See results of regression analyses in Appendix B).

### 3.3. Associations between Perceptions

Pearson correlations were calculated to examine the associations between the study variables. Analysis revealed that physicians who held a positive perception towards the quality of using telephone visits were also likely to have a positive perception towards the safety of the service (*p* < 0.001, r = 0.73) and expressed satisfaction with using telephone visits (*p* < 0.001, r = 0.62). Similarly, physicians who positively perceived the service’s safety were also likely to express satisfaction with using it (*p* < 0.001, r = 0.68).

### 3.4. In-Depth Interviews

The interviews highlighted three themes equivalent to the main survey variables: (1) the quality of the telephone visit service, (2) the safety of the service, and (3) physicians’ satisfaction.

#### 3.4.1. The Quality of the Telephone Visit Service

Most interviewees gave a high rating to the quality of care provided using a telephone visit in cases where there is no need for a physical examination (84.6%). As with the survey responses, family physicians tended to rate the quality of care higher than pediatricians, who expressed more concerns regarding the quality of care provided in telephone consultations. Family physicians mentioned that quality medical care using telephone visits could be provided in cases of older patients, patients with pain management, and chronic diseases. As Interviewee 2, a family doctor, explained: “The telephone visit is suitable for follow-up calls, reporting on treatment, side effects from a drug, conversations about the results of tests, various administrative issues such as forms, chronic problems such as balancing diabetes, medication changes. On the other hand, anything with a physical change requiring an examination is unsuitable for a telephone visit, like a new problem or an existing problem that requires special follow-up”. Most interviewees noted that very few telephone visits end with the patient being invited for an examination at the clinic, and even fewer end with a visit to the emergency department, with the assessment of about 10% of the patients required to arrive at the clinic after a telephone visit.

#### 3.4.2. The Safety of the Telephone Visit Service

Regarding safety, most interviewees noted that patient safety is maintained in telephone visits, as in visits to the clinic (69.2%). However, 46% of the physicians expressed concerns about patient safety, mainly due to the inability to closely examine patients. In addition, physicians noted they feel that communicating with patients during telephone visits is complicated as they cannot see the patient’s reactions or ensure understanding of the information by the patients, which may affect the physicians’ decision making. As Interviewee 4, a family doctor, explained: “I’m afraid of an inaccurate diagnosis and other risks to a patient’s safety, also because I can’t see them. If I could see what the patient looks like, for example listening to the lungs, and more, it would be much better, and I would miss less. Also, regarding explanations about the treatment, it is easier to ensure understanding when they are here. It’s easier to miss information on the phone”. In total, 77% of the interviewees noted that telephone visits are safe only with former patients they have already seen in the clinic. Knowing the patient affects the quality and safety of care provided in a telephone visit. Some interviewees mentioned that in cases where they do not know the patients and there are personal or psychological issues, they prefer that the patient come at least once to a face-to-face visit in the clinic. As Interviewee 9, a family physician explained: “The experience of the doctor is important, but what is of particular significance is gaining a thorough understanding of the patient before beginning the telephone visit. Whether the doctor is experienced or new, does not matter. What matters is a former relationship with the patient”.

#### 3.4.3. Physicians’ Satisfaction

Most interviewees expressed high satisfaction with telephone visits and noted that they are convenient and effective in terms of healthcare delivery when used correctly (77%). Some physicians mentioned the usefulness of using telephone visits and the positive effect on workload. As Interviewee 12, a family doctor, explained: “I perceive the service to be very efficient and easy to use. I am very satisfied. Receiving patients only face-to-face is very exhausting at the end of the day. It is psychologically difficult to receive so many patients in the clinic. It is a positive change in my routine and relieves the burden. In addition, I am also satisfied because my patients are satisfied, especially the young ones”. However, 15% of the physicians expressed dissatisfaction with telephone visits and significant concerns regarding using telephone visits in broad circumstances. “Doing things over the phone is lower quality than the frontal visit. I don’t see the patient. I strongly believe in the importance of the physical relationship between physicians and patients. Physicians are less able to do what is needed on the phone and cannot reach psychosocial issues. In the clinic, I do get to it. The telephone visit reduces my enjoyment of the medical practice”. (Interviewee 7, a family doctor).

## 4. Discussion

The current multi-methods study examined satisfaction, quality, and safety of telephone visits, as perceived by pediatricians and family physicians in Clalit Health Services. Our findings shed light on some of the barriers involving telephone visits and possible solutions. The study demonstrates disagreement among primary physicians as to the quality and safety levels of the telephone consultation modality, although the reported satisfaction was slightly higher.

The telephone modality is convenient and efficient when patients use it in appropriate cases, such as consultations requiring advice about medicine or treatment, follow-up calls, and laboratory test results. Most interviewees noted that very few telephone visits end with the patient being invited for an examination at the clinic, and even fewer end with a visit to the emergency department, with the assessment of about 10% of the patients required to arrive at the clinic after a telephone visit. However, the physicians noted that some cases require a physical examination or a conversation about psychosocial issues in which there is no substitute for a face-to-face meeting with the patients.

The results showed that physicians did not think the quality of care in telephone visits was similar to the quality of care provided in clinic visits. Interviewees mentioned that the quality of the telephone visit could be maintained primarily in cases where a physical examination is not required. In addition, about half of the respondents did not think the telephone visit was entirely safe for patients. Past studies have also identified concerns among physicians about the quality and safety of phone consultations for telemedicine. A systematic review of telephone triage safety in out-of-hours care found that telephone triage was safe for 97% of patients. However, the review showed that ten studies using high-risk simulated patients showed that only 46% were safe [20]. A recent systematic review that explored the barriers and challenges of telemedicine use found that while physicians recognized the potential benefits of telemedicine, they also had concerns about the inability to conduct physical examinations and possible misdiagnoses [21]. The negative impact of telemedicine on the patient–physician relationship and empathic communication was mentioned previously [22,23], as was the difference in the quality of telephone visits, which varies according to the biopsychosocial complexity of the personal visit with the patient [24].

Furthermore, the availability of culturally and linguistically appropriate telehealth services, as well as confidence in electronic health-related communication or care, were factors associated with the adoption of telemedicine [25]. Our findings showed that physicians from the ultra-Orthodox sector expressed lower satisfaction with the telephone modality compared to physicians from the general sector and the Arab sector. A possible reason for this is that the ultra-Orthodox population in Israel shows lower technological skills than the general population and uses telemedicine services less frequently. Differences in perceptions may, in part, emerge from the population that these physicians serve which affects their perceptions. Our findings also showed that physicians who work in the central part of the country expressed lower safety perceptions compared to those in the north and the south of Israel (the periphery). According to the physician interviews, knowing the patient affects the safety of care provided in the telephone visit. A recent study conducted in Israel found that the primary source of treatment preferred by patients is their primary physicians. It was also found that the residents of the periphery gave up medical services significantly more than others [26]. It can be assumed that most of them turn to their primary physician as their first address which creates a better familiarity between the physician and the patient. Health equity considerations for telemedicine must take into account demographic factors and their intersections [25].

Aside from the barriers mentioned, our findings showed that 50% of family physicians and 50% of pediatricians mentioned that they were satisfied with the use of telephone consultations. Recent studies have shown high satisfaction with telemedicine by patients and healthcare providers [27,28,29]. A systematic review of telephone consultations in primary care found that telephone consultations were associated with high levels of patient satisfaction, reduced costs, and improved patient care access [30]. Moreover, recent studies showed that patients and healthcare providers were willing to continue to use remote healthcare as part of their follow-up visits even after the COVID-19 pandemic [31,32,33,34].

Previous studies of physicians’ satisfaction with telemedicine found high satisfaction with telemedicine use among physicians across different specialties, geographic locations, practice locations, and care situations, both for patient care and for consultations with other physicians [35,36,37]. A recent study surveyed primary care physicians’ satisfaction with phone consultations for minor illnesses and injuries. It found that physicians were generally satisfied with the phone consultation modality, which was perceived as a valuable tool for improving patient access to care [36]. Physicians tend to be satisfied with telemedicine if they have input into its development, and administrative support, the technology is reliable and easy to use, and there is adequate reimbursement for its use [38].

### Policy Recommendations for Improving the Telephone Consultation Service

Our findings uncover the barriers to the acceptance of this modality and may help to improve and set guidelines that may positively affect the future quality and safety of the telephone visit service and increase the acceptability of this service. These recommendations include:

1  The gap between reported rates of satisfaction and reported rates of quality and safety of telephone visits requires adopting guidelines for a proper and balanced selection of patients and clinical issues, including performing telephone visits mainly with patients that were previously seen in the clinic and when there is no need for a physical examination. This may enable getting the most out of a telephone visit while minimizing potential quality and safety issues.
2  Implementing technological upgrades to the modality (such as transferring photos and documents and incorporating video recording during the telephone visit) is also supported by a recent study that examined how different forms of remote care may affect the clinical behavior of the physician in remote primary care [39] to improve the quality of the service.
3  Implementing patient education practices—explaining to patients about the telephone visit service, the suitable medical conditions for using the service, and how to communicate with the doctor during the telephone visit while emphasizing the patient’s role in telephone communication.

Our findings regarding the obstacles and recommendations described by the participants provide additional insight into the barriers identified by a previous systematic review of empirical studies on online health consultation. Researchers identified several obstacles categorized as internal and external influences on Home Online Health Consultations (HOHC). Internal factors refer to the users’ behaviors and motivations while using and interacting with the system. These factors include patients’ resistance, poor body language and communication, and negative perceptions of HOHC privacy and security. External factors refer to the system usage and surrounding environment that influence users’ acceptance and use of HOHC services, such as slow internet speed, poor network signal, difficulty with system use, lack of organizational support, and home obstructions [40]. The various factors influencing the adoption of telephone visits as a modality for providing healthcare need a further in-depth examination to uncover obstacles and design targeted strategies to address them.

This study has several limitations. There is a possibility that recall bias may have influenced the findings, and the data should be compared with in-person visits in a more controlled manner. Another limitation is that the researchers wrote a questionnaire not previously tested in other research frameworks. Nevertheless, the questionnaire is valid, and high reliability of the questionnaire items was found. Finally, the response rate to the questionnaire was relatively low, and non-randomized data were analyzed. However, an examination of the study data set showed that respondent and non-respondent groups were similar in demographic variables such as gender, population sector, and geographical area of work. Yet, 43.7% of family doctors and 50.4% of pediatricians in Israel are above the age of 55, while our study sample included 76.6% of family doctors and 79% of pediatricians above the age of 51. The low response rate to surveys among physicians is a well-known challenge, especially with online surveys. Similar recent evaluations of Telemedicine yielded similar low response rates ranging from 4.3% [41] to 20% [42]. Nevertheless, although non-randomized with bias in the age variable, our study data set analysis provides valuable insight into the perceptions toward telephone visits among family and pediatrician doctors.

## 5. Conclusions

Our findings highlight the perceptions of family physicians and pediatricians regarding how to use telephone visits while addressing the risk assessment of the modality of care and its benefits in the possible improvement of health services. Although physicians reported satisfaction, the reported rates of quality (2.27 ± 0.76) and safety (3.02 ± 0.75) of telephone visits among physicians should be further examined. Involving primary physicians in the continuous assessment and improvement of the telephone visit modality will increase quality and safety, as well as raise acceptance of the use of telephone visits among primary care physicians.

## Figures and Tables

**Table 1:** The survey instrument validity assessment.

Subset	Number of Statements	Cronbach’s α	ICC (95% CI) *	*p*-Value
Quality	4	0.759	0.714–0.799	<0.001
Safety	5	0.636	0.570–0.695	<0.001
Satisfaction	4	0.776	0.735–0.812	<0.001

* CI—confidence interval; ICC—intra-class correlation coefficient.

**Table 2:** Demographic characteristics of the survey sample.

Variable		Family (N = 249) N (%)	Pediatricians (N = 93) N (%)	All (N = 342) N (%)
Gender	Male	136 (54.8%)	40 (54.0%)	179 (52.3%)
	Female	112 (45.2%)	51 (56.0%)	163 (47.7%)
Population	General	188 (75.5%)	70 (75.3%)	258 (75.4%)
Sector	Arab	41 (16.4%)	15 (16.1%)	56 (16.4%)
	Ultra-Orthodox	20 (8%)	8 (8.6%)	28 (8.2%)
Geographic	North	64 (25.7%)	24 (25.8%)	88 (25.7%)
Area	Center	149 (59.8%)	51 (54.8%)	200 (58.5%)
	South	36 (14.5%)	18 (19.4)	54 (15.8%)
Age Group	≤40	26 (10.4%)	6 (6.5%)	32 (9.4%)
	41–50	32 (12.9%)	13 (14%)	45 (13.2%)
	51–60	89 (35.7%)	28 (30.1%)	117 (34.2%)
	61–70	91 (36.5%)	31 (33.3%)	122 (35.7%)
	70+	11 (4.4%)	15 (16.1%)	26 (7.6%)

**Table 3:** Research variables mean and standard deviation.

Subset	Family Physicians (N = 249) Mean ± SD	Pediatricians (N = 93) Mean ± SD	All Samples (N = 342) Mean ± SD	*p*-Value between Groups *
Quality	2.26 ± 0.77	2.31 ± 0.74	2.27 ± 0.76	0.597
Safety	3.03 ± 0.76	3.02 ± 0.75	3.03 ± 0.76	0.841
Satisfaction	3.65 ± 0.82	3.69 ± 0.76	3.66 ± 0.80	0.689

* *t*-Test.

**Table 4:** Survey responses distribution.

Statement		Disagree	Neutral	Agree
In my opinion, the quality of care in telephone visits is the same as the quality of care provided in-clinic visits.	Family	104 (41.8%)	87 (34.9%)	58 (23.3%)
	Pediatricians	43 (42.6%)	32 (34.4%)	18 (19.4%)
	All	147 (43%)	119 (34.8%)	76 (22.2%)
In my opinion, a remote telephone visit can replace a face-to-face meeting in most meetings in my clinic.	Family	118 (47.6%)	69 (27.8%)	61 (24.6%)
	Pediatricians	47 (50.5%)	29 (31.2%)	17 (18.3%)
	All	165 (48.4%)	98 (28.7%)	78 (22.9%)
The possibility of transmitting photos and documents during a telephone visit will improve the quality of care provided during the visit.	Family	62 (24.9%)	66 (26.5%)	121 (48.6%)
	Pediatricians	17 (18.3%)	19 (20.4%)	57 (61.3%)
	All	79 (23.1%)	85 (24.9%)	178 (52%)
Incorporating video recording during a telephone visit will improve the quality of the care provided.	Family	81 (32.7%)	69 (27.8%)	98 (39.5%)
	Pediatricians	29 (31.2%)	23 (24.7%)	41 (44.1%)
	All	110 (32.3%)	92 (27%)	139 (40.8%)
In my opinion, a telephone visit shortens the duration of the visit compared to a face-to-face visit in the clinic.	Family	56 (22.6%)	57 (23%)	135 (54.4%)
	Pediatricians	24 (26.1%)	18 (19.6%)	50 (54.3%)
	All	80 (23.5%)	75 (22.1%)	185 (54.4%)
Regarding ongoing care, combining a face-to-face visit to the clinic with a telephone visit is recommended.	Family	21 (8.6%)	31 (12.7%)	192 (78.7%)
	Pediatricians	8 (8.6%)	9 (9.7%)	76 (81.7%)
	All	29 (8.6%)	40 (11.9%)	268 (79.5%)
I think telephone visits do not eliminate the need for face-to-face meetings and are complementary to them.	Family	106 (43.1%)	56 (22.8%)	84 (34.1%)
	Pediatricians	38 (40.9%)	25 (26.9%)	30 (32.3%)
	All	144 (42.5%)	81 (23.9%)	114 (33.6%)
In my opinion, a telephone visit increases the pressure for patients.	Family	188 (76.1%)	43 (17.4%)	16 (6.5%)
	Pediatricians	74 (81.3%)	12 (13.2%)	5 (5.5%)
	All	262 (77.5%)	55 (16.3%)	21 (6.2%)
In my opinion, a telephone visit is safe for the patient and does not involve increased risks.	Family	125 (50.6%)	53 (21.5%)	69 (27.9%)
	Pediatricians	41 (45.1%)	26 (28.6%)	24 (26.4%)
	All	166 (49.1%)	79 (23.4%)	93 (27.5%)
In my opinion, telephone visits harm patient compliance in carrying out the medical recommendations compared to a face-to-face visit at the clinic.	Family	103 (42.6%)	59 (24.4%)	80 (33.1%)
	Pediatricians	46 (49.5%)	25 (26.9%)	22 (23.7%)
	All	149 (44.5%)	84 (25.1%)	102 (30.4%)
The inability to closely examine patients makes the doctor’s decision making more difficult.	Family	56 (22.7%)	71 (28.7%)	120 (48.6%)
	Pediatricians	15 (16.1%)	24 (25.8%)	54 (58.1%)
	All	71 (20.9%)	95 (27.9%)	174 (51.2%)
I sometimes feel uncertain about patients’ medical conditions during a telephone visit.	Family	68 (27.5%)	68 (27.5%)	111 (44.9%)
	Pediatricians	25 (27.2%)	22 (23.9%)	45 (48.9%)
	All	93 (27.4%)	90 (26.5%)	156 (46%)
I feel confident in my professional abilities to perform a telephone visit correctly and optimally.	Family	43 (17.6%)	65 (26.5%)	137 (55.9%)
	Pediatricians	18 (19.4%)	18 (19.4%)	57 (61.3%)
	All	61 (18%)	83 (24.6%)	194 (57.4%)
I am satisfied with the use of telephone visits.	Family	63 (25.6%)	59 (24%)	124 (50.4%)
	Pediatricians	24 (26.1%)	22 (23.9%)	46 (50%)
	All	87 (25.7%)	81 (24%)	170 (50.3%)
Telephone visits are usually conducted efficiently and without technical problems.	Family	46 (19%)	45 (18.6%)	151 (62.4%)
	Pediatricians	13 (14.3%)	24 (26.4%)	54 (59.3%)
	All	59 (17.7%)	69 (20.7%)	205 (61.6%)
In most cases, patients are available during telephone visits and hold a conversation with the doctor attentively and in a quiet environment.	Family	61 (25%)	72 (29.5%)	111 (45.5%)
	Pediatricians	24 (26.1%)	36 (39.1%)	32 (34.8%)
	All	85 (25.3%)	108 (32.1%)	143 (42.6%)
In my opinion, it is safe to conduct a telephone visit only with existing patients whom the doctor has met face-to-face before	Family	39 (16.2%)	52 (21.65)	150 (62.2%)
	Pediatricians	16 (17.2%)	18 (19.4%)	59 (63.4%)
	All	55 (16.5%)	70 (21%)	209 (62.6%)

**Table 5:** Recommended conditions in which telephone visits can be safely used.

Modes of Use	Family Physicians (N = 155) N (%)	Pediatricians (N = 67) N (%)	All Respondents (N = 222) N (%)
A physical examination is not required	23 (14.8%)	4 (6%)	27 (12.2%)
Admin care, routine actions	47 (30.3%)	15 (22.4%)	62 (27.9%)
General and urgent consultations	29 (18.7%)	8 (11.9%)	37 (16.7%)
Follow-up, prescriptions	31 (20%)	16 (23.9%)	47 (21.2%)
Not complicated cases	8 (5.2%)	16 (23.9%)	24 (10.8%)
Parental guidance	0	3 (4.5%)	3 (1.4%)
Referrals	10 (6.5%)	5 (7.5%)	15 (6.8%)
Contagious diseases	7 (4.5%)	0	7 (3.2%)

## Data Availability

The data that support the findings of this study are available from the corresponding author.

## Supplementary Materials

The following supporting information can be downloaded at: https://www.mdpi.com/article/10.3390/healthcare12020212/s1, File S1: Study questionnaire and interview guide.

## Appendix A. Interviewees’ Characteristics

**Table A1:** Interviewees’ Characteristics and coding.

Interview Number	Male/ Female	Specialization	Seniority (Years)
1	F	Family physician	30
2	F	Family physician	15
3	M	Family physician	20
4	F	Family physician	18
5	M	Family physician	20
6	F	Pediatrician	11
7	M	Family physician	15
8	M	Pediatrician	11
9	M	Family physician	5
10	F	Family physician	36
11	F	Family physician	20
12	F	Family physician	17
13	F	Family physician	30
14	F	Family physician	1
15	F	Family physician	25
16	M	Family physician	32
17	M	Family physician	25
18	F	Pediatrician	20
19	M	Family physician	28
20	M	Family physician	35
21	M	Pediatrician	33
22	M	Family physician	25
23	M	Pediatrician	27
24	F	Pediatrician	20
25	F	Pediatrician	20
26	F	Family physician	20

## Appendix B. Regression Analyses

**Table A2:** Regression model for study variables as predictors of perceptions toward quality.

Dimension/Variable	B(SE_B_)	*β*	*p* Value
Gender	−0.13(0.11)	−0.00	0.90
Age	0.54(0.05)	0.59	0.30
Type of Doctor	0.02 (0.12)	0.01	0.85
R^2^	0.07		
F	0.46		

**Table A3:** Multiple regression model for study variables as predictors of perceptions toward safety.

Dimension/Variable	B(SE_B_)	*β*	*p* Value
Gender	−0.02(0.08)	−0.01	0.73
Age	0.04(0.04)	0.65	0.25
Type of Doctor	−0.05(0.09)	−0.03	0.79
R^2^	0.05		
F	0.49		

**Table A4:** Multiple regression model for study variables as predictors of perceptions of satisfaction.

Dimension/Variable	B(SE_B_)	*β*	*p* Value
Gender	−0.01(0.09)	−0.01	0.90
Age	−0.01(0.04)	−0.01	0.30
Type of Doctor	0.03(0.10)	0.01	0.85
R^2^	0.00		
F	0.07		
