# Supplementary material for: Evaluation of Telephone Visits in Primary Care: Satisfaction of Pediatricians and Family Physicians and Their Perceptions of Quality of Care and Safety
Source: Healthcare (Basel). 2024 Jan 15;12(2):212. doi: 10.3390/healthcare12020212 (PMC10815269; doi:10.3390/healthcare12020212)
Supplement: Supplementary file 1 [file healthcare-12-00212-s001.zip › healthcare-2804007-supplementary.pdf]

Supplementary File S1

A. Study questionnaire for physicians

Greetings,

The use of Telemedicine is becoming more prominent in the Israeli healthcare system. This questionnaire is administered as part of a study examining the use of telephone visits in primary care. The questionnaire refers to your own opinions, feelings, and perceptions as they are expressed when providing medical care to your patients using telephone visits. Responding to this questionnaire is voluntary. The survey is anonymous, and the answers will remain confidential. Some statements are written in the masculine tense for convenience but are intended to refer to males and females. We would appreciate it if you would complete the attached questionnaire. It is expected to take about 10 minutes to complete.

For further information, contact Dr. Tamar Shalom via email:

tamar.shalom.100@gmail.com

Part 1: please answer a few general questions for the research purposes:

1. Do you have an experience in telephone visits? 1. Yes 2. No
2. Gender: 1. Male 2. Female
3. Age: _______
4. Number of years of experience as a physician (including residency): ________
5. Geographical area of work in Israel: North, center, South
6. Number of years of experience in telephone consultations with patients: _____
7. Sector of the population: General, Arabic, Ultra-orthodox

Part 2: Circle the number indicating the extent to which you agree with the following statements:

**Statement**	**Strongly disagree**				**Strongly agree**
1. In my opinion, the quality of care in telephone visits is the same as the quality of care provided in-clinic visits.	1	2	3	4	5
2. In my opinion, a remote telephone visit can replace a face-to-face meeting in most meetings in my clinic.	1	2	3	4	5
3. The possibility of transferring photos and documents during the telephone visit will improve the quality of care provided during the visit.	1	2	3	4	5

4.	Incorporating video recording during the telephone visit will improve the quality of the care provided.	1	2	3	4	5
5.	In my opinion, the telephone visit shortens the duration of the visit compared to a face-to-face visit in the clinic.	1	2	3	4	5
6.	As far as ongoing care is concerned, combining a face-to-face visit to the clinic with a telephone visit is recommended.	1	2	3	4	5
7.	I think telephone visits do not eliminate the need for face-to-face meetings and are complementary to them.	1	2	3	4	5
8.	In my opinion, a telephone visit increases the pressure among the patients	1	2	3	4	5
9.	In my opinion, a telephone visit is safe for the patient and does not involve increased risks.	1	2	3	4	5
10.	In my opinion, the telephone visit harms patient compliance in carrying out the medical recommendations, compared to a face-to-face visit at the clinic.	1	2	3	4	5
11.	The inability to closely examine the patient affects the doctor's decision-making and makes it difficult.	1	2	3	4	5
12.	I sometimes feel uncertain about the patient's medical condition during the telephone visit.	1	2	3	4	5
13.	I feel confident in my professional abilities to perform a telephone visit correctly and optimally.	1	2	3	4	5
14.	I am satisfied with the use of telephone visits	1	2	3	4	5
15.	Telephone visits are usually conducted efficiently and without technical problems.	1	2	3	4	5
16.	In most cases, the patients are available by phone during the phone visit and hold the conversation with the doctor attentively and in a quiet environment.	1	2	3	4	5

17. In my opinion, my patients are satisfied with the telephone visit services	1	2	3	4	5
18. In my opinion, it is safe to conduct a telephone visit only with existing patients whom the doctor has met face-to-face before	1	2	3	4	5

Please indicate here the situations you believe telephone visits can be used safely: ____

Please indicate whether you wish to be interviewed regarding your opinions about telephone visits. If you do, please provide contact details: ___________________

B. <u>Study Interview Guide</u>

1. Tell me a little about yourself, what is your profession at the Clalit health services, how many years have you been in your current position?
2. Do you use telephone visits with your patients? How much do you use this modality of care with patients?
3. Do you perceive this modality as convenient, effective?
4. Do you think that providing care through telephone visits is difficult or more complicated compared to face-to-face meeting with patients?
5. Are you afraid of making an inaccurate diagnosis or providing the wrong treatment when giving a telephone consultation? Do you think there are risks to patient safety in telephone visits?
6. Do you feel that providing care through telephone visits affects your decision making or causing uncertainty? How do you deal with it? Can you think of a potential solution for this issue?
7. Do you feel that you must use "intuitive" thinking or decision making when using telephone visits?
8. Do you think that the patients' knowledge or health literacy is significant or affecting your decision making in a different way compared to face-to-face meeting at the clinic?

9. Do you encounter hostility, impatience from patients during telephone consultations compared face-to-face meetings at the clinic?
10. What do you think can be done to improve the user experience of the telephone visit modality?
